# A quality improvement approach to scaling up a complex health system intervention for the prevention and management of cardiovascular disease in rural Indonesia

**DOI:** 10.1371/journal.pgph.0005577

**Published:** 2025-12-04

**Authors:** Thomas Gadsden, Sujarwoto Sujarwoto, Sekar Aqila Salsabilla, Asri Maharani, Devarsetty Praveen, Gindo Tampubolon, Seye Abimbola, Anushka Patel, Anna Palagyi

**Affiliations:** 1 The George Institute for Global Health, University of New South Wales, Sydney, Australia; 2 Faculty of Medicine, University of New South Wales, Sydney, Australia; 3 Department of Public Administration, University of Brawijaya, Malang, Indonesia; 4 Division of Nursing, Midwifery and Social Work, School of Health Sciences, University of Manchester, Manchester Academic Health Science Centre (MAHSC), Manchester, United Kingdom; 5 The George Institute for Global Health, University of New South Wales, Hyderabad, India; 6 Global Development Institute, University of Manchester, Manchester, United Kingdom; 7 National Institute for Health and Care Research (NIHR) Policy Research Unit in Healthy Ageing, University of Manchester, Manchester, United Kingdom; 8 Faculty of Medicine and Health, University of Sydney, Sydney, Australia; University of Embu, KENYA

## Abstract

Scaling up effective public health interventions is crucial for achieving universal health coverage, yet remains challenging. We report the use of the Plan-Do-Study-Act (PDSA) quality improvement model to support the iterative scale-up of a community-based cardiovascular disease risk management program in Malang District, East Java, Indonesia. A pragmatic implementation study comprising three PDSA cycles was conducted in 10 ‘test of scale-up’ villages between April 2021 and December 2022. Each cycle included: 1) the capture of quantitative outcomes, such as the number of new community members screened per month and diagnostic summaries with predicted risk status; and 2) semi-structured interviews and focus group discussions with health care workers, community members, and community health workers in each village to assess acceptability, adoption, adaptations and perceived effectiveness. Based on identified implementation barriers, local Technical Working Groups designed change strategies, which were implemented and evaluated in subsequent cycles. The COVID-19 pandemic disrupted program delivery in the first two PDSA cycles, reducing screening to an average of 112 and 7, respectively. In cycle 3, 463 community members were screened. Across the 10 participating villages, 42 interviews and 30 focus group discussions were conducted per PDSA cycle. Key barriers included difficulty reaching male community members, inadequate resourcing, limited essential medications and poor integration with existing health information systems. Change strategies included centralising screening activities, leveraging instant messaging platforms, additional activities to engage men and streamlined procurement processes. Each village demonstrated versatility in addressing implementation challenges. These findings highlight the utility of the PDSA model in supporting the iterative scale-up of a community-based cardiovascular disease risk management programs in real-world settings, even amid significant disruptions such as the COVID-19 pandemic.

## Introduction

In global public health, the ‘know-do’ gap is a persistent hurdle to improving health outcomes [1]. This term refers to the gap between evidence-based interventions and their implementation in real world settings. Rather than identifying effective health interventions, the challenge lies in scaling and integrating them within complex health systems [2]. Scale-up efforts are influenced by systemic, structural, and contextual factors including resource constraints, health workforce capacity, governance, and socio-cultural, economic, and political dynamics [2,3]. Frameworks to guide the scale up process increasingly recognise that the design and implementation of scale-up efforts must account for and adapt to these factors [4–7].

The ‘Framework for Going to Full Scale’ provides a systematic and adaptive approach to move from small-scale pilot projects to larger, full-scale implementation in diverse and complex environments [8]. Central to this framework are quality improvement collaboratives, which bring together teams from different settings to accelerate process improvement [9]. The Plan-Do-Study-Act (PDSA) cycle drives this process by generating rapid learning about whether an intervention is working in a given context, while identifying and testing changes to enhance effectiveness [10]. PDSA follows a four-stage cycle: in the ‘plan’ stage a change aimed at improvement is identified, the ‘do’ stage tests this change, the ‘study’ stage evaluates its impact, and the ‘act’ stage identifies informs adaptations for the next cycle [11]. PDSA cycles are underpinned by continuous data collection, small-scale testing and iterative hypothesis-driven changes [12].

Although the PDSA method was originally developed for use in clinical settings, it has increasingly been applied in community-based health systems [13]. For example, in South Africa, community health workers delivering maternal and child health services received training and bi-monthly mentoring to review data, identify delivery gaps and test change ideas [14]. Similar projects have been conducted in South Africa, Malawi and Mozambique regarding HIV services [15]; to strengthen referrals and linkages between community health workers (CHWs) and primary health care centres in Ethiopia [16]; and supporting village volunteers in Tanzania and Uganda [17]. However, despite increased use, evidence of effectiveness remains mixed, with recent systematic reviews highlighting limitations in study quality and reporting [18–20].

This study explores the application of PDSA cycles in the preparatory phase of the scale up of a technology-enabled model of care for cardiovascular disease (CVD) risk management (SMART*health*) across Malang District, Indonesia. The SMART*health* program supports the provision of preventive CVD care at the community level by facilitating risk screening for adults aged 40 years and over with referral, tailored clinical decision support for medicines prescription, and active patient follow-up by community health workers (CHWs; locally known as kaders) [21]. The SMART*health* program has been found to be a cost-effective means to increase the use of preventive drug therapies among people with high CVD risk, reduce blood pressure and improve five-year survival rates for people living in rural Indonesia [22–24]. The Malang District Government subsequently committed to investing in the scale up of SMART*health* to the District’s 390 villages (2.5 million target population), including continued collaboration with academic partners to facilitate and evaluate the process of scale up for the first 100 villages.

The scale up of the SMART*health* program provided a unique opportunity to explore the practicalities of embedding the PDSA quality improvement methodology within the expansion of a complex health system intervention to a large population. The primary aim of this research was to harness the PDSA process to devise localised adaptations to enhance the impact and sustainability of the SMART*health* model of care in Malang District and understand how these adaptions could facilitate scale-up.

## Materials and methods

### Study setting and the SMARThealth program

Malang is the second largest district in East Java, Indonesia and, in 2020, had a population of 2.9 million people distributed across 33 sub-districts (*Kecamatan*) and 390 villages (*desa or kelurahan*), of which approximately 70% are rural and 30% urban [25]. This study was conducted across 10 ‘test of scale-up’ villages in Malang (Table 1), selected as being representative of villages taking part in the full SMART*health* scale up. The ten participating villages were located in the catchment of five primary health care centres (*Pusat Kesehatan Masyarakat* or *Puskesmas*) designated for the delivery of CVD referral services, each of which typically serves 25,000–30,000 individuals [26]. They are typically staffed with a general practitioner supported by nurses, midwives and a pharmacist. The Ponkesdes (village health post) serves as an extension of the Puskesmas at the village level, providing basic health services and is staffed by a nurse and midwife [27].

**Table 1:** Health service access in villages participating in the SMART*health ‘*test of scale-up’ [28].

Village	Total population	Catchment Puskesmas (primary health centre)	Distance to Puskesmas (km)	Number of Ponkesdes	Number of Polindes (village maternity post)	Number of pharmacies
Dadapan	7,902	Wajak	5	1	0	0
Jatiguwi	11,934	Sumberpucung	5	1	0	0
Karangduren	8,899	Malang	3	1	1	1
Karangkates	13,807	Sumberpucung	6	1	0	1
Kendalpayak	9,406	Pakisaji	3	1	1	2
Majangtengah	13,979	Dampit	3	0	0	0
Mendalanwangi	7,384	Wagir	3	1	1	2
Sepanjang	16,294	Gondanglegi	5	1	0	0
Sidorahayu	8,909	Wagir	3	1	0	1
Sukolilo	4,039	Malang	5	1	1	0

**Fig 1:**
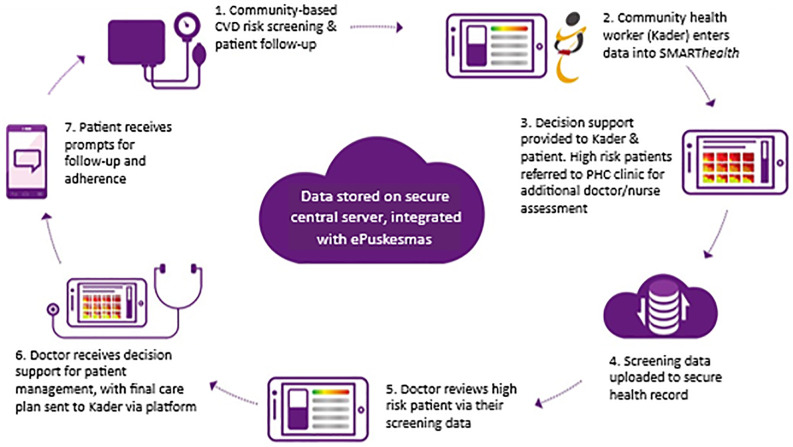
The SMARThealth Cardiovascular Disease cycle of care in Malang District, Indonesia.

To facilitate SMART*health* implementation, kaders conduct monthly screening sessions through the Posbindu, an integrated health service for the early detection and prevention of non-communicable diseases. Kaders are community health volunteers, almost always women, who support health promotion, basic health monitoring, and outreach activities at the village level [29]. All household members aged 40 years or older are invited to participate in a CVD risk assessment using a clinical decision support system on an Android tablet device [21]. During screening, kaders measure body weight, height, blood pressure and blood glucose levels and individual CVD risk is predicted based on World Health Organization Guidelines [30]. Individuals with a predicted high risk of CVD receive a referral to the Puskesmas where clinical assessment measures are repeated and the clinical decision support system for doctors provides recommendations for prevention and management, including medications. As part of the program, nurses also had delegated authority to provide prescriptions for a 30-day supply of medicines (as opposed to the routine 15 days). Kaders then provide monthly follow-up of all high-risk individuals in the community to support lifestyle and medication adherence. All data collected through the SMART*health* application were integrated with the primary health care electronic health information management system (ePuskesmas) and stored on a central server to allow doctors and nurses to view data acquired by kaders and for kaders to view the treatment recommendations made by doctors and nurses. The decision support and patient engagement cycle of care is summarized in Fig 1.

### Study design

**Fig 2:**
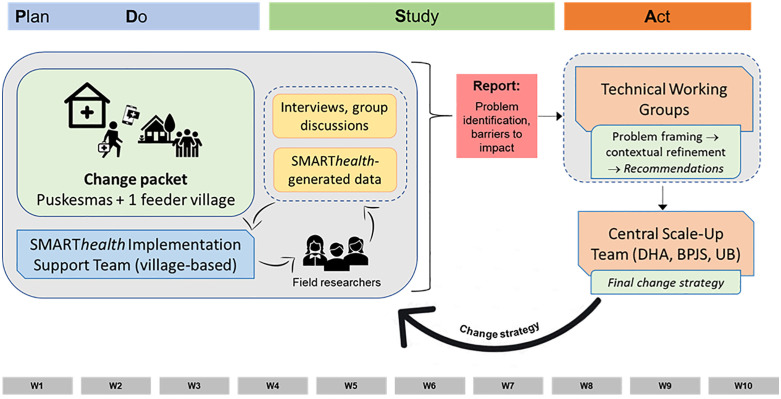
Schematic of PDSA cycle design (cycles 1 and 2).

A pragmatic implementation study was undertaken to test and refine the SMART*health* implementation strategy prior to going to full scale [31,32]. The strategy was iteratively optimised through three PDSA quality improvement cycles conducted across the 10 ‘test of scale-up’ villages. Cycles 1 and 2 were implemented over 10-week periods in 2021, from April to June and June to August, respectively. Each cycle followed a consistent structure: the first 5 weeks focused on planning and implementation (“Plan” and “Do”), followed by 5 weeks of reflection and analysis (“Study”) to identify key implementation challenges and inform adjustments for the next cycle. Insights gathered during the ‘Study’ phase of each cycle directly informed adaptations made during the subsequent ‘Plan’ phase, with many change strategies evolving incrementally across cycles. Cycle 2 coincided with the Delta wave of the COVID-19 pandemic, which led to local lockdowns and restrictions on community activities. Cycle 3 ran over an extended 14-month period (October 2021 to December 2022) due to issues associated with the SMART*health* technology platform. Fig 2 provides a schematic of the PDSA design.

Consistent with the PDSA principle of testing changes on a small scale [8], each village implemented its own set of change strategies under the direction of a local Implementation Support Team, which consisted of the Village Head, primary care nurse, and kader coordinator. These teams were responsible for leading implementing at the village level and liaising regularly with the Malang District Health Authority (DHA).

Technical support was provided by five Technical Working Groups (TWGs), each of which focused on one of five specific implementation enablers: [1] service delivery, [2] workforce management, [3] data and eHealth system integration, [4] medications and equipment, and [5] health promotion. TWGs included DHA department heads (e.g., pharmacy, eHealth/IT, human resources, health promotion, and the NCD unit), as well as a Puskesmas Head and medical doctor from each village. TWGs reviewed implementation data at the end of each PDSA cycle and recommended refinements to address barriers.

A Central Scale-Up Team, comprising representatives from the Malang DHA, BPJS, and the academic implementation partner (University of Brawijaya), reviewed TWG recommendations and determined which refinements would be adopted in the next cycle. These decisions were then implemented by the village-level Implementation Support Teams in the subsequent PDSA cycle.

### Data collection

During the “Study” phase of each PDSA cycle, a combination of quantitative and qualitative data was collected to assess implementation progress and inform subsequent refinements. Quantitative process indicators were extracted from the ePuskesmas platform and included the number of community members screened per month (total and by sex), as well as diagnostic summaries indicating predicted high-risk cardiovascular disease (CVD) status (number and percentage, by sex). To complement these measures, semi-structured interviews and focus group discussions were conducted to explore the acceptability, adoption, and perceived effectiveness of the SMARThealth implementation strategy. These qualitative enquiries were guided by the five pre-defined implementation enablers and additionally sought to capture challenges and successes related to change strategies introduced following each PDSA cycle.

Participants were purposively recruited during each cycle: 15–30 June 2021 (cycle 1), 25 August-16 September 2021 (cycle 2) and 18–25 January 2023 (cycle 3). The same individuals or groups of participants were involved across cycles where possible, with purposive recruitment used initially and supplemented as needed to ensure consistent representation. Interviews included Implementation Support Team members, as well as Puskesmas doctors and pharmacists. In each village, focus group discussions were held with *kader* involved in program delivery and with community members. Discussions were undertaken in Bahasa Indonesia using a semi-structured guide designed to prompt reflection on implementation barriers and facilitators (see S1 File and S2 File). All sessions were audio recorded and transcribed verbatim.

### Data Analysis and Interpretation

Focus group and interview audiotapes were transcribed verbatim immediately following data collection. Transcripts were reviewed and coded by two researchers fluent in Bahasa Indonesia and experienced in qualitative analysis. Barriers and facilitators to effective implementation were identified and categorised according to the five pre-defined implementation enablers. These qualitative insights were triangulated with quantitative process data to provide a more nuanced understanding of the implementation challenges and adaptations across villages and PDSA cycles. Rather than providing an in-depth qualitative or quantitative analysis, this manuscript synthesizes findings across data sources to highlight key implementation learnings and adaptations that informed the iterative scale-up process.

### Ethics statement

Ethics approval for this study was granted by the Health Research Ethics Committee of the Medical Faculty of Brawijaya University (236/EC/KEPK/09/2019) and the University of New South Wales Human Research Ethics Committee (HC190531). Written informed consent was obtained from all participants contributing data to the qualitative activities.

## Results

Findings from the interviews, focus group discussions, and process data were analysed to identify key barriers, enablers and change strategies across the three PDSA cycles. Results are presented below according to the five pre-defined implementation enablers.

Across the three PDSA cycles, 42 interviews and 30 focus group discussions were conducted in the 10 participating villages (Table 2). Interview participants included doctors, nurses, pharmacists, village heads, and kader representatives, with consistent representation across all three cycles. Focus group discussions included kaders and community members in each village. Only one male kader participated, reflecting the predominantly female composition of this workforce. Most kader had completed high school and were primarily engaged in domestic work. Among community members, men were predominantly farmers with an elementary school education, while women were mostly housewives with similar educational backgrounds.

**Table 2:** Number of interview and focus group discussion participants across 10 participating villages, by respondent group and PDSA cycle.

Respondent group	Cycle 1		Cycle 2		Cycle 3		Mean age (years)	Mean Time in Role (years)
	Female	Male	Female	Male	Female	Male		
*Interviews*								
Doctors	5	1	4	2	6	0	29.5	1.9
Pharmacists	5	1	5	1	5	1	28.0	1.8
Nurses	6	4	6	4	6	4	31.8	6.5
Kader representatives	10	0	10	0	10	0	41.8	5.6
Village heads	2	8	2	8	2	8	42.6	8.2
*Focus group participants*								
Kaders	61	1	61	1	61	1	38.4	4.8
Community members	62	72	62	53	59	45	54.6	NA

### Service delivery

During cycles 1 and 2, face-to-face service delivery was severely impacted by the COVID-19 pandemic. In cycle 1, villages adopted various strategies to comply with public health restrictions: some continued neighbourhood screening activities but struggled to mobilise participants; others limited screening to high-risk or vulnerable individuals; and some shifted activities to centralised location such as village halls or Ponkesdes. Household visits, which are a key strategy to expand reach, were not conducted by any village. Another reported barrier at this time was the shortage of doctors and nurses due to their reallocation to the pandemic response.

In cycle 2, as the pandemic worsened, only three villages conducted screening activities. To comply with public health restrictions these took place in centralised locations (rather than household and neighbourhood-based) and only included community members with a known history of comorbidities. However, patients often reported avoiding screening activities due to fear of infection. The pandemic situation eased by cycle 3, allowing most villages to resume household and neighbourhood screening activities. Several factors were highlighted as facilitators of effective implementation in cycle 3: eight villages adopted WhatsApp as a communication channel to promote screening to the community, two villages noted that consistent support and encouragement from the Village Head strengthened relationships between kaders, nurses, doctors and the village government, and five villages reported that kaders resumed door-to-door screenings. Respondents noted that kaders rotated screening locations within the village to boost awareness and coverage. The number of community members screened per village by PDSA cycle is reported in Table 3.

**Table 3:** Number of community members screened per village by PDSA cycle.

Village	Cycle 1: 10 weeks		Cycle 2: 10 weeks		Cycle 3: 14 months	
	Number screened	Number predicted as high-risk (%)	Number screened	Number predicted as high-risk (%)	Number screened	Number predicted as high-risk (%)
Dadapan	93	22 (23.7)	0	0 (0.0)	520	107 (20.6)
Jatiguwi	213	69 (32.4)	10	4 (40.0)	914	194 (21.2)
Karangduren	222	46 (20.7)	4	0 (0.0)	796	76 (9.5)
Karangkates	35	8 (22.9)	0	0 (0.0)	382	92 (24.1)
Kendalpayak	82	31 (37.8)	0	0 (0.0)	140	55 (39.3)
Majangtengah	77	29 (37.7)	58	3 (5.2)	431	117 (27.1)
Mendalanwangi	141	45 (31.9)	0	0 (0.0)	268	69 (25.7)
Sepanjang	65	14 (21.5)	0	0 (0.0)	469	117 (24.9)
Sidorahayu	149	49 (32.9)	0	0 (0.0)	224	60 (26.8)
Sukolilo	38	11 (28.9)	0	0 (0.0)	490	169 (34.5)

**Fig 3:**
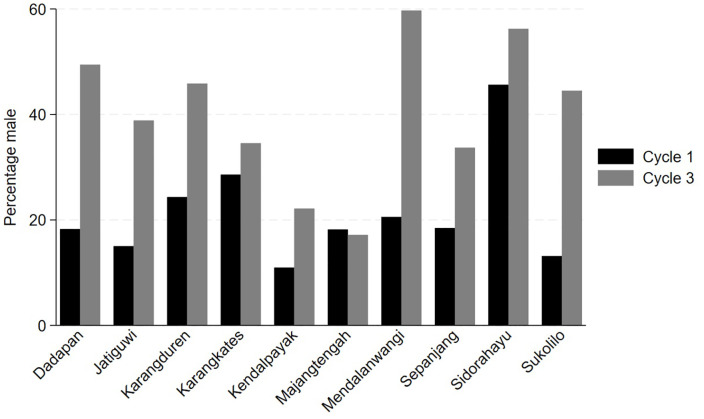
Percentage of males screened in cycles 1 and 3, by village.

Most villages noted that engagement of male community members was limited, as screening events were typically held during work hours. In cycle 1, only 21.3% of newly screened community members were male. Strategies to increase male participation included conducting outreach activities at religious gatherings (Sukolilo) and holding screenings outside of working hours or on weekends (Dadapan and Sidorahayu). By cycle 3, males accounted for 40.2% of screened community members (Fig 3). Additional challenges in cycle 3 included low uptake of referrals to the Puskesmas, often due to distance or limited transportation (Dadapan), kaders and nurses reported receiving insufficient training (Jatiguwi & Karangduren), and kaders not resuming door to door screening but preferring to conduct screening at community events (Karangkates & Sepanjang).

### Workforce management

Many of the service delivery challenges posed by the pandemic affected the health workforce in cycles 1 and 2. The redeployment of health workers during the pandemic significantly reduced health facility hours, limiting patient access. In response, in some villages the local doctor trained village midwives to provide consultation and medication for high-risk CVD community members. This required development of a Standard Operating Procedure and was targeted at senior midwives who had training on NCDs and had worked with the doctor for more than 10 years. Other key barriers related to health worker capacity. In cycle 1, three villages reported that additional kaders were required to meet the patient load, and five villages reported that kaders were not confident to use glucometers and required training. By cycle 2, several villages had conducted training to address these needs: two villages trained nurses to consult and dispense medications, while four trained kaders to glucometer use, health promotion and screening. These were reportedly effective in increasing kaders’ confidence and contributed to a stronger relationship between village health workers.

### Data and eHealth system integration

Technical issues with the SMART*health* application first emerged in cycle 1 and persisted throughout all PDSA cycles. All villages reported long loading times and frequent malfunctions, creating a ‘dual burden’ for kaders: first manually recording screening data on paper, then re-entering it into the application when functional. Combined with poor internet connectivity and limited funding for data packs, these challenges constrained the upload of patient data to the central server. Limited variation in attendees, combined with kaders’ inability to access data recorded in the application, lead to concerns that some individuals were screened multiple times.

Another significant challenge was that data collected via the SMART*health* application could not be synced with the ePuskesmas database. As a result, doctors and nurses could not see who had received a high-risk CVD prediction, nor could they enter data on prescribed treatment, meaning kaders could not see who required follow-up. In response, it was necessary to extract this data from the ePuskesmas system. To overcome these challenges posed by the SMART*health* technology, the DHA released a complementary application for kaders to screen community members for a range of health information (not limited to CVD) and aligned with the ePuskesmas database and government reporting requirements. However, this new application did not calculate a predicted CVD risk rating and did not include clinical decision support for nurses and doctors.

### Medicines and equipment

Several barriers associated with the functionality and availability of medicines, equipment and consumables were reported in cycle 1, including the provision of tablets that were incompatible with the SMARThealth application, delayed procurement of medicines and delivery of consumables and faulty screening equipment. The regulatory change allowing 30-day prescriptions for CVD medicines was rarely implemented as intended. As previously mentioned, the reallocation of health workers to focus on the pandemic response severely restricted the functioning of local health facilities. As a result, lower level facilities that depend on the puskesmas for medication were unable to replenish their stocks. Thus, medicines were commonly prescribed for 3 or 3–7 days only at the Polindes and Ponkesdes, respectively. Despite this, individuals with health insurance (BPJS members) could still obtain 30-day scripts at the Puskesmas, compared to 7–15 days for non-members, and were prioritized access in some cases. In Sepanjang, the Ponkesdes dispensed medicines free of charge to BPJS members in the mornings, while non- members were asked to return in the afternoon to purchase medicines from the nurse’s private clinic.

While some barriers were addressed by cycle 2, such as repairs to screening equipment, it was not until cycle 3 that villages reported the procurement of new tablets. While all villages reported greater financial support from the village government by cycle 3, medicine stocks remained limited and prescription of short scripts continued. Villages adopted various practices to expand access to medicines. For instance, in Karangduren and Karangkates, patients attending screening were required to bring plastic waste which kaders would later sell and use the proceeds to purchase consumables. In Majangtengah, health workers purchased medicines privately and were then reimbursed by community members. Community members reportedly preferred this, as it avoided the cost of travelling to the Puskesmas. Wealthier community members typically purchased medicines at private clinics where, if not covered by BPJS, they would pay out of pocket. Another commonly reported barrier was that high-risk individuals often do not take up their referral to the Puskesmas. This was typically attributed to distance and cost of transport, or fear of being diagnosed with a health condition and the associated stigma.

### Health promotion

In cycle 1, only four villages conducted face-to-face health promotion activities for high-risk patients and noted that attendance was low. In cycle 2, only Sukolilo conducted face-to-face health promotion. was conducted. In response to these barriers, health promotion activities shifted to social media and WhatsApp. The latter was widely used as kaders leveraged the existence of village WhatsApp groups to disseminate information and videos, initially on the topics of healthy diets and medication adherence and later on COVID-19 prevention. WhatsApp was also used for one-on-one communication with high-risk individuals and allowed kaders to stay up to date with their condition and share information regarding the availability of medication. By cycle 3, health promotion activities were resumed in neighbourhoods and conducted alongside events to promote attendance such as aerobics exercise sessions. In two villages, kaders reportedly lacked confidence in delivering health promotion activities, so nurses conducted the sessions. In another village, residents requested that doctors, not kaders, lead health promotion sessions. Attendees were primarily older, less-educated women from lower-income households. Efforts to attract others included holding health promotion activities outside of working and school hours and during weekends.

**Fig 4:**
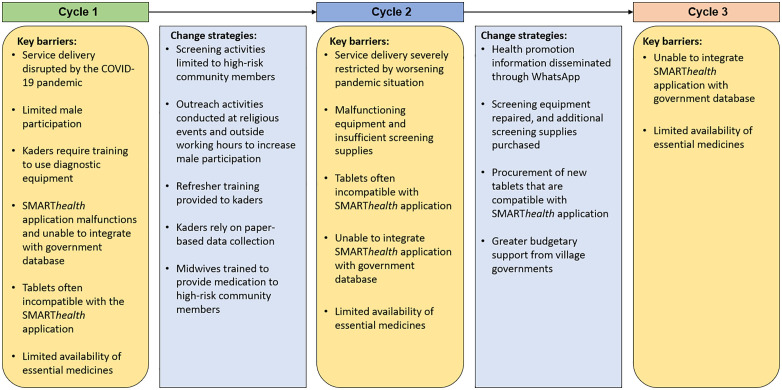
Driver diagram and key change strategies for all participating villages.

Fig 4 presents a driver diagram that summarises the key findings across the PDSA cycles and illustrates the change ideas tested.

## Discussion

This study provides a unique account of applying the PDSA quality improvement process to scale up of a complex health system intervention for community-based prevention and management of CVD. The study demonstrated strong buy-in from key stakeholders, including local health workers and community members, which served as a critical facilitator of implementation. However, the unforeseen impact of the COVID-19 pandemic and persistent issues related to the SMART*health* application hindered implementation as intended. Nonetheless, iterative adaptations to the implementation strategy across the three cycles progressively addressed barriers and enhanced program delivery. In the context of limited research on quality improvement processes in complex health system interventions, this study provides valuable insights and can serve as a blueprint for other scale-up efforts. These findings will also inform the optimisation of the SMART*health* program in the next phase as it moves to full-scale implementation in Malang district.

Recent reviews of quality improvement initiatives in primary health care highlight facilitators including the co-development of a clear scale-up strategy, support from dedicated implementation teams, strong buy-in across health system levels and the use of proven interventions [9,33,34]. This study reflects many of these enablers while underscoring the unique importance of strong commitment from local village governments to the test of scale up. Indeed, each village government continued to address implementation barriers by providing additional resources and equipment despite the disruption caused by the COVID-19 pandemic. Such leadership is increasingly recognised as critical to the success of quality improvement initiatives [18,33,34] and, considering Indonesia’s hierarchical local governance structures, likely played an important role in fostering broader stakeholder engagement [35].

While the use of the PDSA model has been limited in Indonesia, our findings support its potential to engage kaders in leading localised quality improvement efforts. This aligns with evidence in sub-Saharan Africa, where PDSA has empowered community health workers to adapt and scale interventions to their contexts [15,20,36]. For example, in Uganda, a PDSA-based initiative improved family planning service delivery by CHWs by empowering them to lead the local adaptation of the intervention in each site [37]. Similarly, in Tanzania and Uganda, village volunteers were trained to use PDSA cycles to create, test, and scale up change ideas to improve uptake of maternal and newborn health services [17].

The implementation of the test of scale-up was constrained by intractable issues related to the digital technology. Specifically, the SMART*health* application’s sub-optimal performance and its inability to integrate with the existing electronic health information system (ePuskesmas) persisted through all cycles. These challenges significantly increased the data collection burden on kaders, necessitating paper-based recording that may have introduced errors. Additionally, the lack of data integration prevented doctors from accessing screening data or recording treatment decisions for kaders to follow up on, compromising a key program component (i.e., kader follow-up of community members to ensure treatment adherence). These findings highlight the need for robust, interoperable digital health systems to support quality improvement efforts at scale.

Contextual influences further shaped implementation outcomes. Beyond COVID-19 disruptions, socio-cultural norms such as gender roles in rural Indonesia influenced program delivery. Men, who are typically the primary income earners and often engaged in agricultural work, were under-represented in screening activities, a finding which was also evident in the original SMART*health* trial [22,38]. Successful change strategies to increase male participation, such as screening outside working hours and at diverse locations, should inform future efforts. These findings underscore the importance of culturally sensitive adaptations in quality improvement initiatives.

A major strength of this study was the integration of quantitative screening data with qualitative insights from interviews and focus groups, providing a rich understanding of both outcomes and stakeholder experiences throughout the implementation process. The longitudinal collection of qualitative data across cycles enabled iterative learning and responsive adaptations. Conversely, unavoidable limitations included the COVID-19 pandemic’s significant disruption to the first two cycles, restricting our ability to fully evaluate the intervention’s initial impact. Additionally, the extended duration of cycle 3 (14 months vs. the intended 10 weeks) introduced variability that may have affected comparability but it also reflects the realities of adaptive implementation in complex settings.

## Conclusion

This study demonstrates the utility of the PDSA quality improvement model in supporting the iterative scale-up of a community-based CVD risk management program in a real-world setting, even amidst significant disruptions such as the COVID-19 pandemic. The capacity to flexibly adapt intervention delivery models in response to local implementation barriers, while engaging stakeholders in co-developing solutions, highlights PDSA as a valuable tool for scaling complex health system interventions. These insights can guide policymakers and implementers aiming to scale similar interventions in dynamic and resource-constrained environments.

## Supporting information

S1 File PDSA Interview template. (DOCX)

S2 File PDSA Focus Group Discussion template. (DOCX)

S1 Checklist Inclusivity in global research. (DOCX)
