# Supplementary material for: A quality improvement approach to scaling up a complex health system intervention for the prevention and management of cardiovascular disease in rural Indonesia
Source: PLOS Glob Public Health. 2025 Dec 4;5(12):e0005577. doi: 10.1371/journal.pgph.0005577 (PMC12677765; doi:10.1371/journal.pgph.0005577)
Supplement: S1 File — (DOCX) [file pgph.0005577.s001.docx]

**Supplementary File 1: PDSA Interview template**

Brief introduction (Facilitator)

*We are undertaking an assessment of the SMARThealth program in your village during the period from [start of PDSA cycle] to now. Today, we are interested to learn from your experience of the SMARThealth program during this time. The information you provide will be used to identify the parts of the SMARThealth program that work well, and the parts that can be made stronger to ensure that the ongoing implementation of SMARThealth is effective, efficient and equitable.*

General Perception

1. In your opinion, what has worked well with the SMARThealth program so far, and what has worked not so well?

Program Improvement

1. Are you aware of any changes in the way services of the SMARThealth program are delivered since the last time we spoke?

If yes:

- 1. Can you tell me about these changes? What are they and why were they implemented?
  2. In your opinion, have these strategies improved service provision?
     - If yes, in what way have they improved the SMARThealth program?
     - If no, why do you think they have not worked? In what way has COVID-19 influenced implementation?

Service Delivery

1. a) What worked well and what problems were experienced by kaders in the screening of patients for CVD risk in your village?

b) Are you aware of any strategies used by the kaders to make sure they screened all the patients in their assigned area?

1. Are you aware of any barriers that stopped patients from attending the ponkesdes or puskesmas to take up their referral to see the nurse/doctor or replenish their medicines supply? What factors do you think help patients to attend the ponkesdes or puskesmas?

Workforce Management

1. a) As a member of the SMARThealth Implementation Support Team, do you feel confident in your ability to monitor the SMARThealth program implementation in your village and deal with any problems? What, if any, additional training or support to you think you need to help you to confidently support SMARThealth implementation into the future?

b) Do you feel that the SMARThealth Implementation Support Team provides kaders with adequate direction and support to help them feel confident and motivated in their work in the SMARThealth program?

c) In what ways does the SMARThealth Implementation Support Team work well together and in what ways could your working relationship be strengthened?

1. What kind of support do you personally provide the kaders in your village? How do you think they can be best supported to provide quality health care for the community?
2. Do you feel that the kaders have received adequate training provide them with the knowledge and skills they need to implement the SMARThealth program in a high quality manner? If no, please tell me what additional training kaders’ need.
3. Is the village leadership supportive of the SMARThealth program?

- If yes, can you talk a little about the type of support they provide to make SMARThealth a success in your village? *[Prompts: encouragement; advocacy; budget allocation; supervision]*

- If no, what additional support do you feel kaders need from the village leadership?

Data & eHealth System Integration Strategy

1. a) Are you aware of any challenges experienced by kaders with the use of the SMARThealth technology? If yes, please tell me more about these challenges.

b) Have you been able to use the SMARThealth platform to monitor the work of the kaders in your village?

- If yes, tell me how you have used the SMARThealth platform.

- If no, how did you monitor the work of the kaders?

c) What parts of the SMARThealth tablet application do you think could be improved to help you perform your work?

Medications and Equipment

1. a) To your knowledge, did your village have adequate equipment and consumables for kaders to be able to perform their screening activities, and for the nurses to run their cardiovascular disease clinics at the ponkesdes or posbindu? Tell me about any issues you are aware of in relation to equipment or consumables.

b) Are you aware of any barriers to the supply and access of medicines for high risk CVD patients identified in the SMARThealth program? Can you talk about these.

Health Promotion Activities

1. a) To your knowledge, how did patients and the broader community engage with the health promotion aspects of the SMARThealth program?

b) Which community members were more likely to engage with the health promotion aspects of the program? [Prompt: wealthier/poorer, younger/older, more/less educated, men/women, natives/migrants].

c) What do you think would make people in the community more likely to participate in the health promotion activities which are part of the SMARThealth program? What have you done to encourage people to participate?

1. Finally, is there anything else you would like to comment on about the SMARThealth program in your village?
