# Supplementary material for: A quality improvement approach to scaling up a complex health system intervention for the prevention and management of cardiovascular disease in rural Indonesia
Source: PLOS Glob Public Health. 2025 Dec 4;5(12):e0005577. doi: 10.1371/journal.pgph.0005577 (PMC12677765; doi:10.1371/journal.pgph.0005577)
Supplement: S2 File — (DOCX) [file pgph.0005577.s002.docx]

**Supplementary File 2: PDSA Focus Group Discussion template**

Brief introduction (Facilitator)

*We are undertaking an assessment of the SMARThealth program in [insert village] during the period from [start of PDSA cycle] to now. Today, we are interested to learn from your experience of the SMARThealth program during this time. The information you provide will be used to identify the parts of the SMARThealth program that work well, and the parts that can be made stronger to ensure that the ongoing implementation of SMARThealth is effective, efficient and equitable.*

General Perception

1. Please tell me about your experience with the SMARThealth the program.

Program Adaptation/COVID

1. Can you describe how COVID-19 has affected the way you use the health services?

Please think about:

- have you been able to see the kader, nurse or doctor when you needed?
- have you been able to uptake referral to see the doctor?
- have you been able to access medicines? If yes, how? If no, why not?
- are people in the community still getting health care when they need it (for non-COVID related conditions)?

Service Delivery

1. a) How often did the kaders make a visit to screen or follow up patients in the community?

b) Where did these visits occur (in your own home, in another location)? Where would you prefer to see the kader?

c) Describe the parts of the SMARThealth program in your neighbourhood that you think work well and the parts that did not work so well.How could the SMARThealth program be improved?

1. a) Did the kader provide you with any information about cardiovascular disease and its prevention using the SMARThealth technology?

- If yes, please tell me the information you remember being provided by the kader.

- If no, were you provided with information about cardiovascular disease and its prevention by the kader in another way? Please describe this.

1. Were there any barriers to patients being able to take up their referral to the nurse or doctor at the ponkesdes or puskesmas? Can you talk about these?

Medications and Equipment

1. a) If you were prescribed medicines, did you experience any issues with being able to access these?If yes, can you talk about these problems?

b) Where did you go to get your medicines?

c) What were the reasons why some patients did not collect their medicines supply?

[Prompts: difficult to access the posbindu/ponkesdes location; no medicines in stock; clinic time not suitable; didn’t see importance of continuing medicines; fearful of seeing the nurse (or doctor)]

Health Promotion Activities

1. a) Did you or your family members participate in the health promotion aspects of the program?

b) In your opinion, did the health promotion influence community to take up healthy behaviours? Tell me more about this – in what way did these activities influence your behaviour? Which of these activities did you find most informative for learning about healthy behaviour?

c) Which community members were more likely to engage with the health promotion aspects of the program? [Prompt: wealthier/poorer, younger/older, more/less educated, men/women, natives/migrants].

d) What do you think would make people in the community more likely to participate in the health promotion activities which are part of the SMARThealth program?

Workforce Management

1. Tell me about your relationship with the kader in your neighbourhood. What do you think of the work that they do? Do you trust the health care and information they provide you?
2. Tell me about your relationship with the nurses at the ponkesdes and posbindu, and the doctor in your village.Do you feel comfortable to visit the nurse or doctor if you need to? Do you trust the care and information they provide you with? Are there certain things that make you more or less likely to visit the nurse or doctor for an appointment (please tell me more about these)?
